# A generalized model for time-resolved luminescence of localized carriers and applications: Dispersive thermodynamics of localized carriers

**DOI:** 10.1038/s41598-017-00065-3

**Published:** 2017-02-02

**Authors:** Zhicheng Su, Shijie Xu

**Affiliations:** Department of Physics, Shenzhen Institute of Research and Innovation (SIRI), and HKU-CAS Joint Laboratory on New Materials, The University of Hong Kong, Pokfulam Road, Hong Kong, China

## Abstract

For excited carriers or electron-hole coupling pairs (excitons) in disordered crystals, they may localize and broadly distribute within energy space first, and then experience radiative recombination and thermal transfer (i.e., non-radiative recombination via multi-phonon process) processes till they eventually return to their ground states. It has been known for a very long time that the time dynamics of these elementary excitations is energy dependent or dispersive. However, theoretical treatments to the problem are notoriously difficult. Here, we develop an analytical generalized model for temperature dependent time-resolved luminescence, which is capable of giving a quantitative description of dispersive carrier dynamics in a wide temperature range. The two effective luminescence and nonradiative recombination lifetimes of localized elementary excitations were mathematically derived as ${{\boldsymbol{\tau }}}_{{\boldsymbol{L}}}{\boldsymbol{=}}\frac{{{\boldsymbol{\tau }}}_{{\boldsymbol{r}}}}{{\bf{1}}{\boldsymbol{+}}\tfrac{{{\boldsymbol{\tau }}}_{{\boldsymbol{r}}}}{{{\boldsymbol{\tau }}}_{{\boldsymbol{t}}{\boldsymbol{r}}}}({\bf{1}}{\boldsymbol{-}}{{\boldsymbol{\gamma }}}_{{\boldsymbol{c}}}){{\boldsymbol{e}}}^{({\boldsymbol{E}}{\boldsymbol{-}}{{\boldsymbol{E}}}_{{\boldsymbol{a}}}){\boldsymbol{/}}{{\boldsymbol{k}}}_{{\boldsymbol{B}}}{\boldsymbol{T}}}}$ and ${{\boldsymbol{\tau }}}_{{\boldsymbol{n}}{\boldsymbol{r}}}{\boldsymbol{=}}\frac{{{\boldsymbol{\tau }}}_{{\boldsymbol{t}}{\boldsymbol{r}}}}{({\bf{1}}{\boldsymbol{-}}{{\boldsymbol{\gamma }}}_{{\boldsymbol{c}}})}{{\boldsymbol{e}}}^{{\boldsymbol{-}}({\boldsymbol{E}}{\boldsymbol{-}}{{\boldsymbol{E}}}_{{\boldsymbol{a}}}){\boldsymbol{/}}{{\boldsymbol{k}}}_{{\boldsymbol{B}}}{\boldsymbol{T}}}$, respectively. The model is successfully applied to quantitatively interpret the time-resolved luminescence data of several material systems, showing its universality and accuracy.

## Introduction

Carrier localization (CL) in real crystalline solids due to various disorders, e.g., defects, impurities, composition fluctuation, lattice distortion etc. is a ubiquitous phenomenon which was theoretically treated by Anderson for the first time^1^. To date, CL and related phenomena still remain as a subject of extensive interest primarily because of their scientific significance and profound impact on electrical, magnetic and optical properties of material systems^2–18^. With the rapid development of the InGaN alloy based blue-green light emitting diodes, recently, the CL effect induced by structural imperfections has been increasingly addressed^19–21^. For example, it has been well shown that localized carriers due to alloy disorder, especially indium content fluctuation, can produce efficient luminescence and unusual thermodynamic behaviors^19–28^.

In order to interpret these unusual luminescence behaviors associated with the carrier localization, many attempts have been devoted. For example, Eliseev *et al.* proposed an empirical formula to interpret temperature-induced “blue” shift in peak position of luminescence^25^. This model agrees well with experimental data at high temperatures, but does not work at low temperatures. Wang applied the pseudopotential approach to study the CL mechanism in different InGaN systems^23^, which mainly focuses on the contribution of component fluctuation and quantum-dot formation to the carrier localization. No temperature effect was taken into consideration in Wang’s theoretical work. Dal Don *et al.* employed Monte Carlo simulation method to simulate temperature dependent behaviors of peak width and peak position of PL in ZnCdSe quantum islands^29^. Li *et al.* developed an analytical model for steady-state PL of localized state ensemble^22,30,31^. By solving a rate equation taking into account several fundamental processes of localized carriers, they obtained an analytical distribution function for localized carriers and then built up a steady-state luminescence model. They named their formula set LSE (localized-state ensemble) luminescence model^30^. The model not only quantitatively reproduces S-shape temperature dependence of PL peak for different material systems^32^, but also interprets V-shape temperature dependence of PL width and even temperature dependence of integrated luminescence intensity^30,31^. Moreover, It was also proved that LSE model can be reduced to Eliseev *et al.*’s band-tail model at high temperatures^22^. Li and Xu also proved that the integrated luminescence intensity formula of LSE model can be reduced to the well-known thermal quenching formula when the distribution parameter of localized states approaches zero^31,33^. Nonetheless, the model is only applicable for steady-state luminescence of localized carriers, and it does not work for transient or time-resolved luminescence of localized carriers at all. Göbel *et al.* have done a good attempt to quantitative analysis of the time-resolved luminescence process in quantum well structures by numerically solving the rate-equation^34^. But in their work temperature was not taken into consideration. To the best of our knowledge, an analytical model with well-defined physical quantities has not yet been established for temperature dependent time-resolved photoluminescence of localized carriers.

In this article, we attempt to fill the void by developing an analytical model for time-resolved photoluminescence of localized carriers with a substantial energy distribution. As derived and argued below, the model was formulated with two effective luminescence lifetime and nonradiative recombination lifetime. It was then applied to quantitatively interpret the experimental time-resolved luminescence data obtained by several groups, which enables us get deep insight into the recombination dynamics of localized carriers in real material systems.

**Figure 1:**
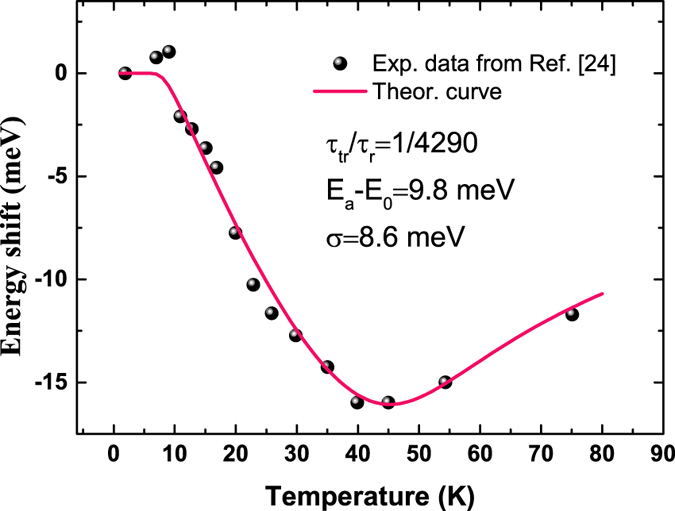
Temperature induced luminescence peak shift of localized carriers in an InGaN/GaN quantum well. The experimental data (solid circles) were measured by Schömig *et al.* ^24^, whereas the solid line was a best fitting curve with Eqs (3) and (4).

For a luminescent system with a total density of states (DOS) of localized electronic states, *ρ*(*E*), time evolution of the excited carrier concentration *N*(*E, T, t*) may be described by a partial differential equation^22,30,35^

1 $$\frac{\partial N(E,T,t)}{\partial t}=G+{\gamma }_{c}\frac{N^{\prime} }{{\rm{\Lambda }}}\rho (E)-\frac{N(E,T,t)}{{\tau }_{tr}}{e}^{(E-{E}_{a})/{k}_{B}T}-\frac{N(E,T,t)}{{\tau }_{r}},$$

where *G*, *γ*_*c*_, *N*′, and Λ represent the generation (excitation) rate of carriers due to optical excitation, electrical injection etc., the re-capture coefficient of the thermally activated carriers, the total number of thermally activated carriers, and the total number of localized electronic states, respectively. In Eq. (1), *E*_*a*_ stands for a distinct energetic position of materials, e.g., the location of a delocalized level to which the localized carriers can be thermally activated^30,31^. Depending on material, *τ*_*tr*_ and *τ*_*r*_ are the two time constants characterizing the thermal activation and radiative recombination processes of carriers, respectively. The latter process produces luminescence. Under the steady-state conditions, i.e., ∂*N*/∂*t* = 0, one can get one solution described by^30^

2 $$N(E,T)=A(T)\cdot f(E,T)\cdot \rho (E),$$

where $f(E,T)=\frac{1}{{e}^{(E-{E}_{a})/{k}_{B}T}+{\tau }_{tr}/{\tau }_{r}}$ represents a distribution function for localized carriers. The explicit expression of *A*(*T*) can be found in our previous publication^30^. As argued previously by us, the lineshape of the steady-state luminescence spectrum of localized states, given by *N*(*E*, *T*)/*τ*_*r*_, is essentially described by *f*(*E*, *T*) · *ρ*(*E*). Under such circumstances, the peak position of the steady-state luminescence of localized states can be found by the following equation set^22,30,31^

3 $$E(T)={E}_{0}-\xi (T)\cdot {k}_{B}T,$$

4 $$[\frac{{\rm{1}}}{\xi (T)}{(\frac{\sigma }{{k}_{B}T})}^{2}-{\rm{1}}]{e}^{-\xi (T)}=\frac{{\tau }_{{\rm{tr}}}}{{\tau }_{{\rm{r}}}}\times {e}^{-({E}_{0}-{E}_{a})/{k}_{B}T},$$

where, *E*_0_ and σ are the parameters derived from $\rho (E)={\rho }_{0}{e}^{-{(E-{E}_{0})}^{2}/2{\sigma }^{2}}$, e.g., a standard Gaussian DOS for localized states. By using above equation set and taking into account the temperature induced bandgap shrinking usually described by Varshni’s empirical formula for ideal semiconductors, we can well reproduce the S-shape temperature dependence of the steady-state luminescence of localized carriers in different materials^32^. Here, let us show an example of application of the model to the experimental data obtained by Schömig *et al.* in an InGaN/GaN quantum well sample^24^. From Fig. 1, it can be seen that the experimental data was nearly perfectly reproduced by the steady-state LSE luminescence model.

As addressed earlier, we are primarily interested in the solution of rate equation under transient conditions, e.g., pulsed optical or electrical excitation, for building up an analytical model for time-resolved luminescence of localized carriers in the present study. To develop such an analytical model, we need to do some analysis to some physical processes, i.e. the re-capture of already-thermally-activated carriers by localized states, and undertake necessary approximation. In the steady-state LSE model, the number of re-captured carriers per unit time is described by ${\gamma }_{c}N^{\prime} \frac{\rho (E)}{{\rm{\Lambda }}}$, where $N^{\prime} ={\int }_{-\infty }^{+\infty }\frac{N(E\text{'},T,t)}{{\tau }_{tr}}{e}^{({E}^{^{\prime} }-{E}_{a})/{k}_{B}T}dE^{\prime}$ represents the total number of thermally activated carriers. Please be noted that the re-capture rate or efficiency is assumed to be a constant for all localized states in the steady-state LSE model^22,30,31^. However, such assumption may be no longer well justified for the localized state system under the transient excitation conditions (i.e., pulsed optical or electrical excitation) because of high carrier density at instant. It is obviously more reasonable to assume that the re-capture rate is a function of localized state energy^36,37^.

In the present study we thus assume that the re-capture efficiency is proportional to the number of unoccupied localized states left by carriers which are thermally activated away, as expressed by

5 $$\eta =\frac{N(E,T,t){e}^{(E-{E}_{a})/{k}_{B}T}}{{\int }_{-\infty }^{+\infty }N(E^{\prime} ,T,t){e}^{({E}^{^{\prime} }-{E}_{a})/{k}_{B}T}dE^{\prime} }.$$

Then the number of re-captured carriers per unit time can be described by

6 $$\frac{\partial {N}_{rc}}{\partial t}={\gamma }_{c}N^{\prime} \eta ={\gamma }_{c}\frac{N}{{\tau }_{tr}}{e}^{(E-{E}_{a})/{k}_{B}T}.$$

Under such an assumption and the transient excitation conditions, the rate equation may be re-written as

7 $$\frac{\partial N(E,T,t)}{\partial t}=G(E,t)-(1-{\gamma }_{c})\frac{N(E,T,t)}{{\tau }_{tr}}{e}^{(E-{E}_{a})/{k}_{B}T}-\frac{N(E,T,t)}{{\tau }_{r}}.$$

Eq. (7) can be simplified as

8 $$\frac{\partial N(E,T,t)}{\partial t}+P(E,T)N(E,T,t)=G(E,t),$$

where

9 $$P(E,T)=(1-{\gamma }_{c}){e}^{\beta (E-{E}_{a})}/{\tau }_{tr}+1/{\tau }_{r},$$

and

10 $$G(E,t)=g(t)\rho (E).$$

Here *β* = 1/*k*_*B*_ *T*. Under such circumstance, a solution of Eq. (8) can be found as

11 $$N(E,T,t)=\rho (E){e}^{-P(E,T)\cdot t}\int g(t){e}^{P(E,T)\cdot t}dt.$$

If the excitation is continuous and constant, i.e., the optical excitation or injection current was kept a constant for time, *g*(*t*) = *g*_0_, then

12 $$N(E,T,t)=\frac{{\tau }_{tr}{g}_{0}\rho (E)}{(1-{\gamma }_{c}){e}^{\beta (E-{E}_{a})}+{\tau }_{tr}/{\tau }_{r}}.$$

By setting *γ*_*c*_ = 0, Eq. (12) is reduced to the steady-state solution described by Eq. (2).

It is well known that in time-resolved luminescence measurements, pulsed excitation was used. For a pulsed excitation, time-dependent creation of carriers may be mathematically stated as^36^

13 $$g(t)={g}_{0}{e}^{\frac{-{(t-{t}_{0})}^{2}}{2{{\sigma }_{t}}^{2}}}.$$

Here *σ*_*t*_ is an important parameter governing the generation process of carriers. Under such pulsed excitation, a solution of Eq. (8) may be written as

14 $$N(E,T,t)=\sqrt{\frac{\pi }{2}}{\sigma }_{t}{g}_{0}\rho (E)\{{\rm{erf}}\,[\frac{t-({t}_{0}+{\sigma }_{t}^{2}P(E,T))}{\sqrt{2}{\sigma }_{t}}]+1\}{e}^{{\sigma }_{t}^{2}P{(E,T)}^{2}/2+({t}_{0}-t)P(E,T)}$$

Then, time evolution of luminescence intensity of localized carriers may be formulated as

15 $${I}_{L}(E,T,t)=\frac{N(E,T,t)}{{\tau }_{r}}\propto \rho (E)\cdot \{{\rm{erf}}\,[\frac{(t-{t}_{0})-{\sigma }_{t}^{2}/{\tau }_{L}}{\sqrt{2}{\sigma }_{t}}]+1\}{e}^{-(t-{t}_{0})/{\tau }_{L}},$$

where

16 $${\tau }_{L}(E,T)=\frac{1}{P(E,T)}=\frac{{\tau }_{r}}{1+\tfrac{{\tau }_{r}}{{\tau }_{tr}}(1-{\gamma }_{c}){e}^{(E-{E}_{a})/{k}_{B}T}}.$$

In fact, Eq. (16) can be simplified as

17 $${\tau }_{L}=\frac{{\tau }_{r}}{1+{e}^{\alpha (E-{E}_{m})}},$$

which is widely adopted in literature^37–39^. Here, *E*_*m*_ was defined by Oueslati *et al.* as a specific energy at which the recombination rate equals the transfer rate, and *α* was a model dependent parameter with the unit of reverse energy^38^. Note that our model takes into account the temperature effect. In other words, Eq. (16) gives a quantitative description of dispersive thermodynamics of localized carriers for luminescence.

**Figure 2:**
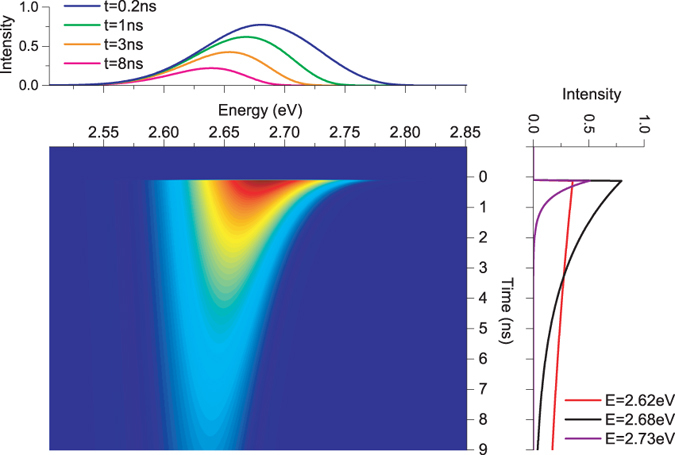
Calculated TRPL spectra (image) of a localized state system with Eq. (15). Obviously, the theoretical luminescence lifetime shows a distinct dependence on energy (right figure), while luminescence spectrum exhibits an interesting dependence on the delay time (top figure).

By adopting parameters of *σ*_*t*_ = 1.5 *ps*, *γ*_*c*_ = 0.148, *τ*_*tr*_ = 0.625 *ns*, *τ*_*r*_ = 16.557 *ns*, *E*_*a*_ = 2.718 *eV*, *E*_0_ = 2.684 *eV*, *σ* = 50 *meV* and *T* = 300 *K*, we calculate time-resolved PL (TRPL) spectra of a localized state system with Eq. (15). The calculated TRPL spectra are illustrated in a two-dimensional image in Fig. 2. The horizontal axis of the image stands for energy while the vertical axis represents delay time. The image contrast displays luminescence intensity, i.e., red color means strong emission. The top figure shows several theoretical PL spectra at different delay times, whereas the right figure depicts three luminescence intensity decaying traces for three different photon energies. Obviously, the theoretical TRPL spectra based on Eq. (15) exhibit interesting evolution tendency upon delay time. For example, luminescence lifetime shows a distinct dependence on energy and luminescence spectrum exhibits an interesting dependence on the delay time, i.e., fast redshift of the peak position at an early stage of the delay time and rapid narrowing of the lineshape at higher energy.

It is well known that the measured luminescence time constant is usually written as ${\tau }_{L}^{-1}={\tau }_{nr}^{-1}+{\tau }_{r}^{-1}$. By using this relationship and Eq. (16), we can derive an explicit expression of *τ*_*nr*_:

18 $${\tau }_{nr}(E,T)=\frac{{\tau }_{tr}}{(1-{\gamma }_{c})}{e}^{-(E-{E}_{a})/{k}_{B}T}.$$

**Figure 3:**
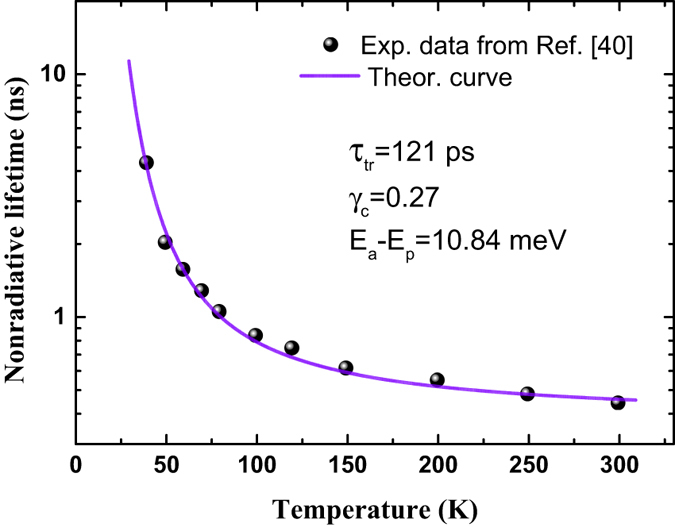
Temperature dependence (solid circles) of the nonradiative lifetime for localized excitons of InGaN MQWs, adopted from ref. 40. The solid line represents a best fitting with Eq. (18).

Above physical quantity may be regarded as an effective nonradiative recombination time of localized carriers. Eq. (18) tells us that the effective nonradiative recombination time of localized carriers exhibits a distinct exponential dependence on temperature and energy, predominantly governing the luminescence lifetime. The temperature dependence of nonradiative lifetime for localized excitons of InGaN MQWs, measured by Narukawa *et al.* ^40^, can be quantitatively interpreted with Eq. (18), as shown in Fig. 3.

**Figure 4:**
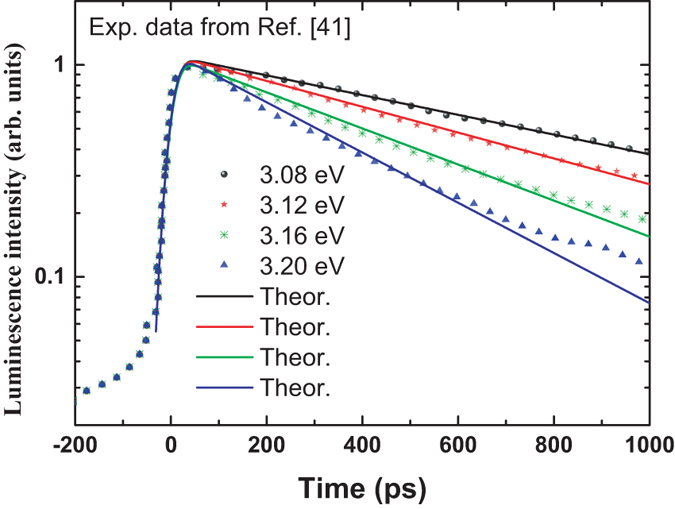
Time-resolved photoluminescence traces (various solid symbols) of a single-layer InGaN alloy measured by Satake *et al.* for different energy^41^. The solid lines are the fitting curves with Eq. (15).

Now we apply the model developed in the present work to the time-resolved photoluminescence traces of a single-layer InGaN alloy measured by Satake *et al.* ^41^. In such InGaN ternary alloy, localized excitons were proposed to give a major spontaneous emission^20,41^. In Fig. 4, various symbols represent the experimental data from ref. 41, while the solid lines are the theoretical curves with Eq. (15). Parameters used in the calculation are listed in Table 1. The rise time keeps almost constant of *τ*_*rise*_ ≈ 2.5631*σ*_*t*_ = 49.80 *ps*, while the decay time decreases as the luminescence energy increases. From Fig. 4, its can be seen that very good agreement between theory and experiment is achieved.

**Table 1:** Parameters for the solid curves in Fig. 4.

*E*(eV)	3.08	3.12	3.16	3.20
*τ*_*L*_(*ps*)	932.91	712.54	510.19	366.48
*σ*_*t*_(*ps*)	19.49	19.42	19.43	18.99

**Figure 5:**
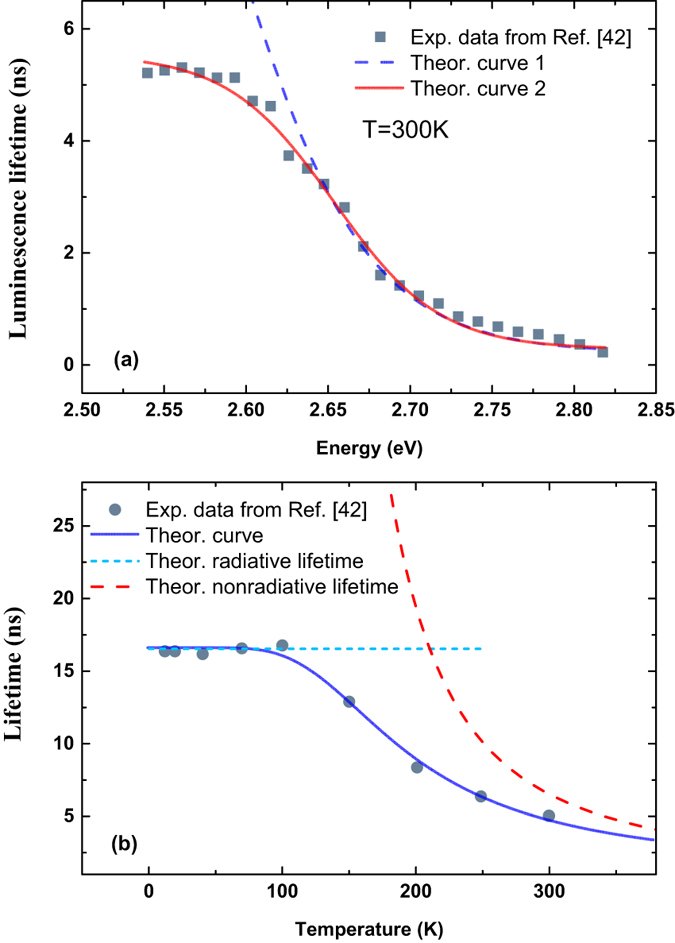
Various lifetimes (solid symbols) of localized carriers in Ag-coated InGaN/GaN quantum wells measured by Okamoto *et al.* ^42^ (**a**) for different energy at room temperature and (**b**) for different temperatures. The solid lines are the calculated results with Eqs (16) and (18), independently or jointly, while the dashed lines represent the theoretical curves for overall luminescence lifetime with different parameters (**a**) and the nonradiative lifetime (**b**) respectively. The short dashed line in (**b**) indicates constant radiative lifetime.

In addition to InGaN alloy, InGaN/GaN quantum wells, which usually act as the active layers of blue/green LED’s^19^, also exhibit strong localization effect. Okamoto *et al.* experimentally measured the PL lifetimes of localized carriers in Ag-coated InGaN/GaN quantum wells for different energy and different temperatures^42^. The solid symbols in Fig. 5(a) and (b) are their experimental data. For the luminescence lifetimes, one can see that they exhibit an interesting dependence on energy. In Fig. 5(a), solid and dashed lines are two theoretical curves with Eq. (16). For the solid line, parameters of *γ*_*c*_ = 0.148, *E*_*a*_ = 2.718 *eV*, *τ*_*tr*_ = 0.625 *ns* and *τ*_*r*_ = 5.273 *ns* were adopted, while parameters of *γ*_*c*_ = 0.148, *E*_*a*_ = 2.718 *eV*, *τ*_*tr*_ = 0.625 *ns* and *τ*_*r*_ = 16.557 *ns* for the dashed curve. Obviously, Okamoto *et al.*’s experimental data can be well reproduced with Eq. (16) when the first set of parameters was adopted. For the second set of parameters, agreement between theory and experiment becomes unideal, especially in the lower energy region. The key reason causing this deviation between theory and experiment in the low energy region is that *τ*_r_ in the second set of parameter is much larger. However, when this value is adopted, temperature dependence of the average luminescence lifetime of localized carriers can be clearly elucidated, as shown by the solid line in Fig. 5(b). That is, the luminescence lifetime is predominantly determined by constant radiative lifetime (*τ*_*r*_ = 16.557 *ns*) for low temperatures <100 K, while it is gradually controlled by temperature-dependent nonradiative lifetime for higher temperatures. It shall be noted that in Fig. 5(a), the dispersive luminescence lifetimes *τ*_*L*_ (i.e., energy dependent) was measured at room temperature (~300 K), whereas the average luminescence lifetimes for all interested energy were measured at different temperatures in Fig. 5(b). For localized carriers, their radiative recombination lifetime usually does not change with temperature in low temperature region^40^. At high temperatures, *τ*_*nr*_ decreases significantly and predominantly determines the luminescence lifetime *τ*_*L*_. In Okamoto *et al.*’s experiment, plasmonic effect of Ag coating thin layer may be a strong function of temperature, which causes very different radiative recombination lifetimes of localied carriers. That is why different values of radiative recombination time *τ*_*r*_ should be used to reproduce the energy dependence of luminescence lifetime *τ*_*L*_.

**Figure 6:**
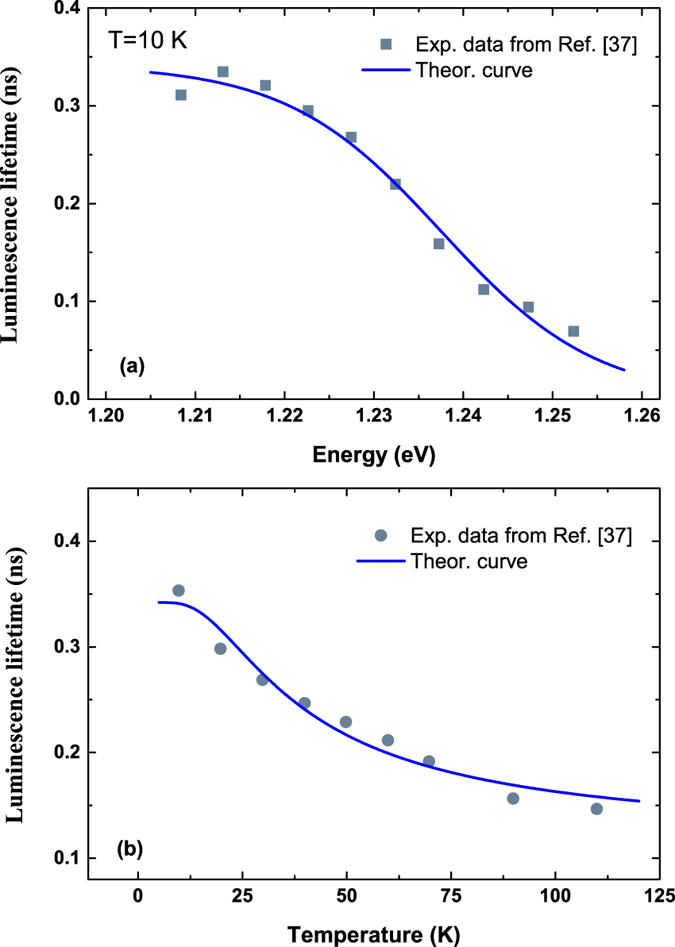
Luminescence lifetimes (solid symbols) of InGaAsN epilayer measured by Mair *et al.* for (**a**) different energy and (**b**) different temperatures^37^. The solid lines are the theoretical curves with Eq. (16).

In addition to InGaN/GaN materails system, this model is also capable of giving quantitative interpretation to the experiemntal data reported by different groups for very different materials with localization effect, such as InGaAsN^37^, InAs QDs^43^ and GaInP^44^. As shown in Fig. 6, for example, the temperature and energy dependent recombination dynamics of InGaAsN epilayer can be well fitted with Eq. (16) using the parameters of *γ*_*c*_ = 0.220, *E*_*a*_ = 1.244 *eV*, *τ*_*tr*_ = 0.129 *ns*, and *τ*_r_ = 0.342 *ns*. Again, excellent agreement between experiment and theory is achieved.

In conclusion, an analytical generalized model for time-resolved luminescence of localized carriers is developed, which is capable of giving quantitative description of dispersive thermodynamics of localized carriers. The explicit expressions of effective luminescence lifetime and nonradiative recombination lifetime of localized carriers were derived for the analytical model. The formulas were used to quantitatively interpret temperature- and energy-dependent time-resolved luminescence data measured by several groups. The model and its applications enable us obtain state-of-the-art understanding of time-resolved luminescence processes of localized carriers, especially temperature and energy dependence of these dynamic processes in semiconductors.
